# Predicting the need for intubation within 3 h in the neonatal intensive care unit using a multimodal deep neural network

**DOI:** 10.1038/s41598-023-33353-2

**Published:** 2023-04-17

**Authors:** Jueng-Eun Im, Seung Park, Yoo-Jin Kim, Shin Ae Yoon, Ji Hyuk Lee

**Affiliations:** 1 Biomedical Engineering, Chungbuk National University Hospital, Cheongju, Republic of Korea; 2 Department of Pediatrics, Chungbuk National University Hospital, Chungbuk National University College of Medicine, Chungdae-ro 1, Seowon-gu, Cheongju, 28644 Republic of Korea

**Keywords:** Health care, Medical research

## Abstract

Respiratory distress is a common chief complaint in neonates admitted to the neonatal intensive care unit. Despite the increasing use of non-invasive ventilation in neonates with respiratory difficulty, some of them require advanced airway support. Delayed intubation is associated with increased morbidity, particularly in urgent unplanned cases. Early and accurate prediction of the need for intubation may provide more time for preparation and increase safety margins by avoiding the late intubation at high-risk infants. This study aimed to predict the need for intubation within 3 h in neonates initially managed with non-invasive ventilation for respiratory distress during the first 48 h of life using a multimodal deep neural network. We developed a multimodal deep neural network model to simultaneously analyze four time-series data collected at 1-h intervals and 19 variables including demographic, physiological and laboratory parameters. Evaluating the dataset of 128 neonates with respiratory distress who underwent non-invasive ventilation, our model achieved an area under the curve of 0.917, sensitivity of 85.2%, and specificity of 89.2%. These findings demonstrate promising results for the multimodal model in predicting neonatal intubation within 3 h.

## Introduction

Respiratory distress is the most common indication for admission to the neonatal intensive care unit (NICU)^1,2^. Endotracheal intubation is the end-stage of respiratory support and is a critical procedure in neonates with respiratory difficulties. Recent non-invasive ventilator (NIV) strategies have reduced the incidence of endotracheal intubation and duration of mechanical ventilator support in NICU management^3,4^. While the increasing use of NIV includes high-flow nasal cannula (HFNC), nasal continuous positive airway pressure (NCPAP), bilevel positive airway pressure (BIPAP), and non-invasive positive pressure ventilation (NIPPV) in neonates with respiratory difficulties, a significant proportion fail on NIV support and require intubation within the first few days of birth, especially in preterm infants^5–7^.

Neonatal respiratory distress syndrome (RDS) is a major source of morbidity in NICU. Ordinary treatments include mechanical ventilation and surfactant replacement therapy^8,9^. Considering the lower incidence of RDS in late preterm and term neonates, it is difficult to distinguish RDS from other less severe respiratory diseases that do not require endotracheal intubation^10^. Although several risk factors for RDS have been established, such as prematurity, cesarean section, perinatal asphyxia, male sex, maternal diabetes mellitus, and multiple births^11–13^, intubation time is often delayed in late preterm and term neonates with respiratory distress receiving NIV support.

Recently, deep neural networks have been widely implemented in neonatal medicine. Examples include a predictive model of mortality during NICU hospitalization^14^ and prediction of long-term neurodevelopmental outcomes at the corrected age of 2 years^15^ using electronic medical records including demographics, vital signs, and images. Previous studies have proposed predictive models for RDS and NCPAP failure using clinical and laboratory parameters in both adult and neonatal medicine^11,16–19^. During the early neonatal period, according to the success of the adaptation to extra-uterine environments, neonates’ cardiopulmonary status is vulnerable and fluctuating. Because the NIV failure commonly occurred in the first-hour stabilization period^20,21^, short-term prediction has practical use in NICU settings. In this study, we designed a multimodal deep neural network (MDNN) model to predict the need for intubation within the next 3 h in neonates with respiratory difficulty who were admitted to the NICU within the first 48 h of life and initially received NIV support. This model is intended to support clinical decisions by providing diagnostic alternatives to physicians and proposing appropriate treatments based on demographic, bedside clinical, and laboratory parameters at the time of NICU admission.

## Methods and materials

### Ethics statement

Data collection was approved by the Institutional Review Board of the Chungbuk National University Hospital (IRB No. 2021-02-034). The review board waived the requirement for informed consent, owing to the retrospective design of this study. We confirm that all methods were performed in accordance with the relevant guidelines and regulations.

### Study population

We retrospectively obtained datasets of all neonates who were admitted to the NICU within the first 48 h of life at Chungbuk National University Hospital between June 1, 2020, and November 30, 2021. We excluded neonates without respiratory problems, those hospitalized after 48 h of life, and those intubated at the time of admission. To improve model performance, we excluded neonates intubated 12 h after admission and those with missing data, defined as more than two tabular data or ≥ 10% of time-series data.

### Datasets

Demographic data, physiological parameters, and laboratory data were collected. The datasets comprised 19 tabular and 4 time-series features. The tabular data in this study are defined as either categorical or numerical variables. We collected data such as gestational age (GA), birth weight, Apgar scores at 1 and 5 min, sex, delivery mode, antenatal steroid use, pregnancy-induced hypertension, gestational diabetes mellitus, premature membrane rupture, birth place, multiple births, initial body temperature, clinical risk index for babies (CRIB-II) score^20^, and parameters in the initial blood gas analysis, including pH, PO_2_, PCO_2_, base excess (BE), and lactate as the tabular features. Additionally, we analyzed four time-series features: heart rate (HR), respiratory rate (RR), fraction of inspired oxygen (FiO_2_), and pulse oximetry (SpO_2_). Time-series data were recorded at 1-h intervals until 12 h after admission. The missing values in the tabular data and time-series data were filled with the average values and the most recent data, respectively.

The time of intubation was defined as the first record of endotracheal intubation or ventilation data. We classified infants with intubation time ≤ 12 h of NICU admission as intubated patients and all others as non-intubated patients. Since the initial tabular data such as body temperature and blood gas analysis results (pH, PCO_2_, PO_2_, BE, and lactate) could gradually recover or worsen over time, it has limitations to provide long-term (> 12 h) predictions. Instead, we focused on alleviating the model’s complexity and improving its practical use by using tabular data, so our model was designed to predict the need for short-term (≤ 12 h) intubation.

**Figure 1:**
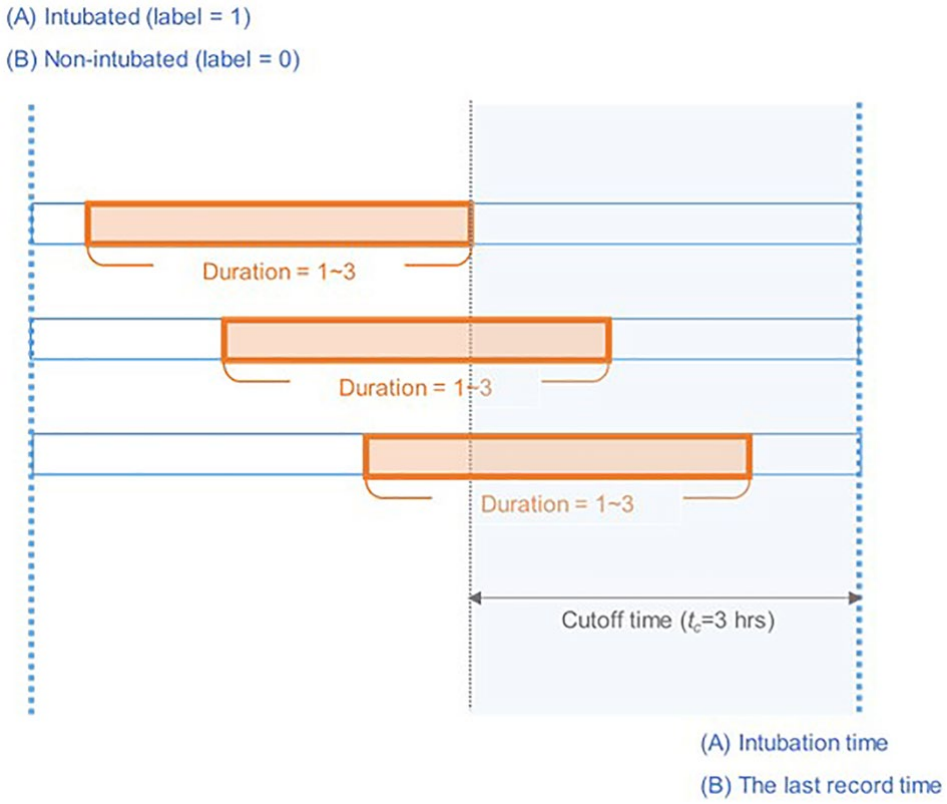
Detailed illustration of dataset generation. The records for individual patients were divided into training and test dataset, and intubated and non-intubated patients were labeled 1 and 0, respectively. Multiple samples were extracted with a random duration (1–3 h) containing the cutoff time (${t}_{c}$), which corresponded to the fixed time before the intubation time or last record time.

Multiple samples were generated from each patient over time. To classify intubation cases 3 h in advance, the samples taken within the *cutoff time* (*t*_*c*_ = 3 h) are labeled as “1” for intubated patients and “0” for non-intubated patients (Fig. 1). For the model training and test, the patients were first split into training and test sets as shown in Supplementary Table S1, multiple samples were then generated from each patient set over time. Therefore, the samples from one patient do not go to both training and test sets. It was inevitable to extract multiple samples from a patient, dividing the entire time sequence by a specific sequence length to balance negative and positive data. For positive data collected prior to intubation attempts, the overall time sequence range was varied from 1 to 12, but the negative data included all time points during 12 h period after admission. We considered that the difference in sequence length could lead to biased results in the model training. In addition, most intubation occurred within 3 h (29/36), so we cut the entire time sequence into the same time sequence length of 3.

Since the aim of this study was to provide decision support for clinicians while assessing the need for intubation upon NICU admission, we limited our prediction time window to within the first 12 h of NICU hospitalization.

### Models

**Figure 2:**
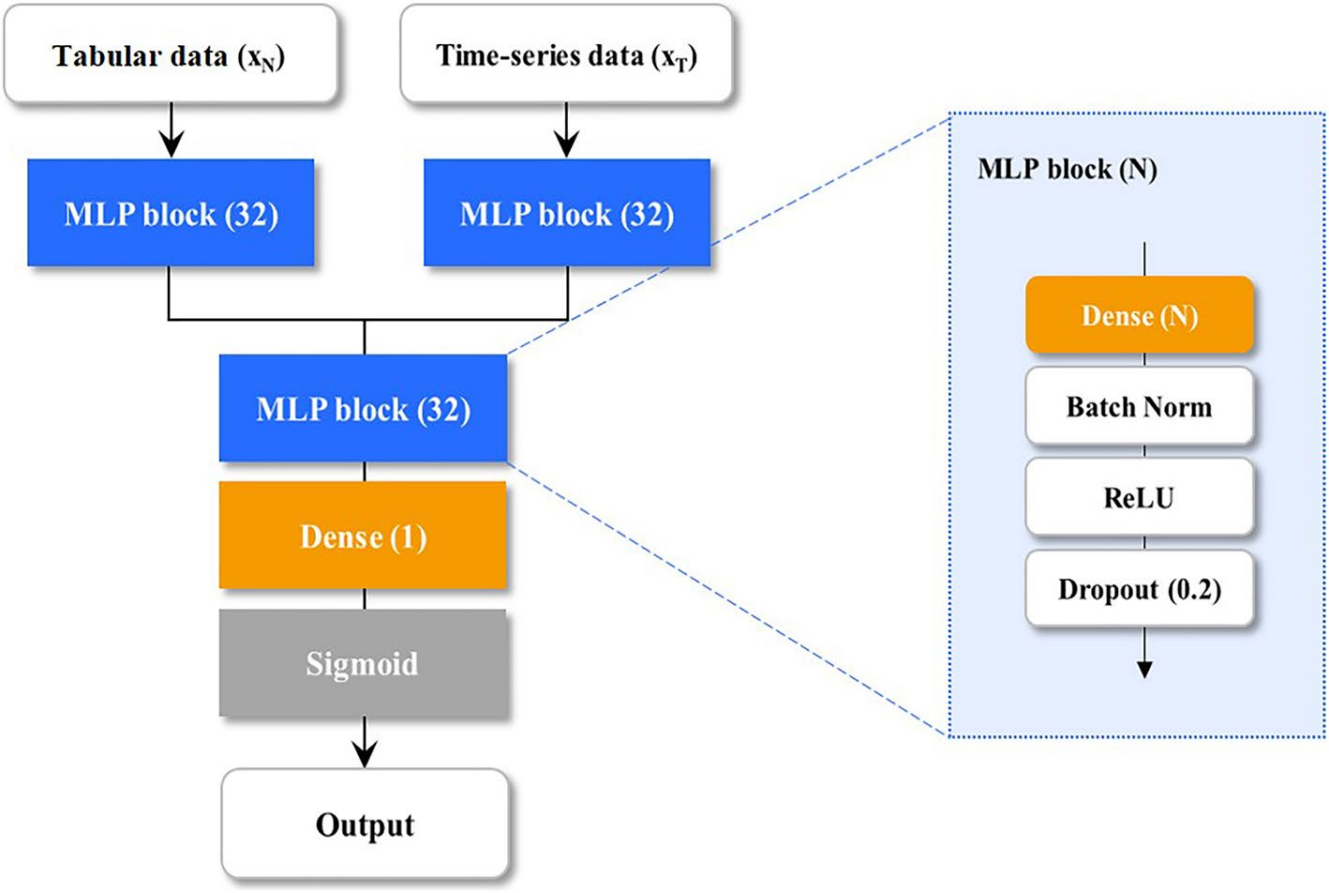
Multimodal deep neural network architecture. The model utilizes two types of input data: tabular and time-series data. These data are analyzed by multilayer perceptron (MLP) blocks, and the outputs of these MLP blocks are concatenated into a feature vector to predict the need for intubation.

We designed an MDNN using three subnetworks to jointly analyze the tabular *x*_*n*_ ${\in {\mathbb{R}}}^{a}$ and time-series data *x*_*t*_ ${\in {\mathbb{R}}}^{b\times l}$ as shown in Fig. 2, where *a* and *b* are denoted as the feature numbers of tabular and time-series data. Subscript *l* indicates the length of the time sequence. First, *x*_*t*_ is flattened, then the flattened *x*_*t*_ and x_n_ are fed into the multilayer perceptron (MLP) blocks, consisting of a single fully connected layer with *d* (= 32) nodes, batch normalization, and rectified linear units, followed by a dropout to alleviate overfitting. The vectors from the MLP blocks are concatenated, and the concatenated vector ${\mathrm{x}}_{\mathrm{cat}}\in {\mathbb{R}}^{2\mathrm{d}}$ is analyzed using the last MLP block. Finally, the analyzed vector was used to calculate the intubation probability (0–1) by the fully connected layer with sigmoid activation. The proposed MDNN was implemented using TensorFlow 2.4 (https://www.tensorflow.org/).

To compare the MDNN with widely used machine learning (ML) methods, we further implemented linear regression (LR), support vector machine (SVM)^21^, and an extreme gradient boosting decision tree (XGBoost) regressor^22^. The SVM is a supervised machine learning algorithm based on kernel functions, and we employed the Gaussian radial basis function for model predictions. The XGBoost model is also considered a supervised technique that ensembles decision trees using the gradient boosting framework. The LR, SVM, and XGBoost are open-source library, and we programmed them using Python scikit-learn 1.0.2 (https://scikit-learn.org/) and XGBoost 1.7.2 (https://xgboost.ai/) libraries.

### Statistical analysis

Continuous variables were compared using the Student’s *t*-test or the Mann–Whitney U test and are presented as the mean (95% confidence interval). Categorical variables were compared using the chi-square test or Fisher’s exact test and are presented as percentages and frequencies. SPSS version 25 (SPSS Inc., Chicago, IL, USA) was used for all statistical analyses, and* P* < 0.05 was considered statistically significant.

### Model evaluation

The models were internally validated using fourfold cross-validation to assess performance and minimize overfitting (Supplementary Table S1). The total datasets were split into training (75%) and test (25%) sets by maintaining the overall positive/negative ratio. The proportion of positive patients in the training and test datasets was set to approximately 30%, the same as in the overall dataset (36 positive patients out of 128 patients).

For each fold, we evaluated five quantitative measures, including the area under the receiver operating characteristic curve (AUROC), F1-score, sensitivity, specificity, and accuracy. The AUROC and F1-score metrics were considered the highest priority because we need to use robust metrics against imbalanced dataset. The F1-score calculates the harmonized mean between precision and recall, and AUROC is calculated from the ROC graph that visualizes the tradeoff between true positive rate and false positive rate. Statistical calculations were performed using the Scikit-learn library (https://scikit-learn.org/)^23^.

## Results

### Baseline demographics

**Figure 3:**
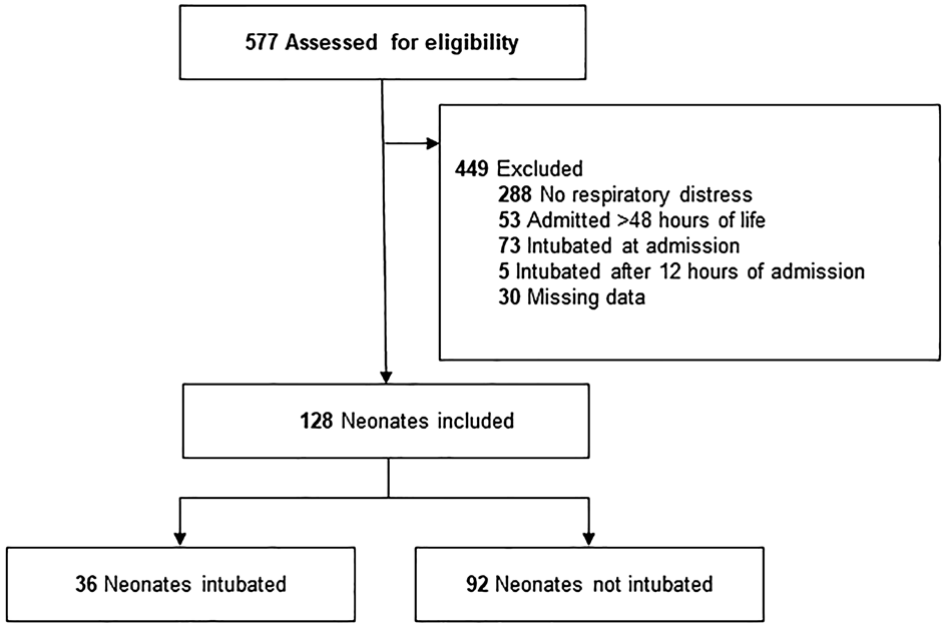
Flow chart for data selection.

Of the 577 neonates admitted to the NICU during the study period, we excluded 449 who did not meet the inclusion criteria and 30 with missing data. The datasets included 128 eligible neonates with 36 intubated (positive) and 92 non-intubated patients (negative) (Fig. 3). The mean GA and birth weight were 35.8 ± 2.8 (30–42) weeks and 2.6 ± 0.8 (0.9–4.9) kg, respectively. Table 1 shows the clinical characteristics of the intubation and non-intubation groups.

**Table 1:** Baseline characteristics and outcomes of cohort.

Variable	Intubated (n = 36)	Non-intubated (n = 92)	*P*-value
Gestational age, mean (CI), week	35.2 (34.3‒36.1)	36.1 (35.5‒36.7)	0.127
Preterm, no. (%)	20 (56)	48 (52)	0.844
Birth weight, mean (CI), kg	2.6 (2.3‒2.8)	2.6 (2.4‒2.8)	0.736
Male sex, no. (%)	23 (64)	49 (53)	0.325
1-min Apgar score, mean (CI)	8.6 (8.2‒9.0)	8.8 (8.6‒9.0)	0.225
5-min Apgar score, mean (CI)	9.5 (9.2‒9.8)	9.7 (9.6‒9.8)	0.201
Cesarean delivery, no. (%)	26 (72)	62 (67)	0.675
Inborn, no. (%)	22 (61)	66 (72)	0.290
Multiple birth, no. (%)	9 (25)	19 (21)	0.637
Antenatal steroids use, no. (%)	11 (31)	33 (38)	0.539
Premature membrane rupture, no. (%)	9 (25)	20 (22)	0.823
Maternal hypertension, no. (%)	3 (8)	6 (7)	0.710
Maternal diabetes mellitus, no. (%)	4 (11)	9 (10)	0.757
CRIB II score, mean (CI)	3.1 (2.7‒3.5)	2.9 (2.6‒3.1)	0.382
NIV type			
HFNC, no. (%)	22 (61)	79 (86)	0.003*
NCPAP or BIPAP, no. (%)	14 (39)	13 (14)	
Initial blood gas analysis			
pH, mean (CI)	7.2 (7.2‒7.2)	7.3 (7.2‒7.3)	0.004*
PCO_2_, mean (CI), mmHg	63.4 (59.5‒67.4)	57.2 (55.4‒59.1)	0.001*
PO_2_, mean (CI), mmHg	37.1 (33.7‒40.5)	40.5 (36.9‒44.2)	0.273
Base excess, mean (CI), mmol/L	−5.3 (−6.1 to −4.6)	−4.4 (−5.0 to −3.9)	0.075
Lactate, mean (CI), mmol/L	2.7 (2.24‒3.21)	3.4 (3.0‒3.8)	0.081
Heart rate at admission, mean (CI), bpm	143.5 (140.3‒146.6)	133.9 (133.04‒134.7)	<0.001*
Respiratory rate at admission, mean (CI), breath/min	50.8 (46.9‒54.7)	51.7 (50.6‒52.9)	0.651
Fraction of inspired oxygen at admission, mean (CI), %	27.3 (25.8‒29.0)	22.6 (22.5‒22.8)	< 0.001*
Pulse oximetry at admission, mean (CI), %	93.3 (91.9‒94.8)	97.2 (97.0‒97.4)	< 0.001*
Body temperature at admission, mean (CI), °C	36.2 (36.0‒36.3)	36.1 (36.0‒36.2)	0.470
Time to intubation, mean (CI), min	124 (120)	–	–

*CI* 95% confidence intervals, *HFNC* high-flow nasal cannula, *NCPAP* continuous nasal cannula airway pressure, *BIPAP* bi-level positive airway pressure.

*P-value < 0.05.

In the initial blood analysis results of the intubated group, average pH was considerably lower (*P* = 0.004) and PCO_2_ was higher (*P* = 0.001) than those of the non-intubated group. Of the 128 neonates who initially received NIV support, 22 of 101 (22%) infants primarily supported by HFNC and 14 of 27 (52%) infants initially treated with NCPAP or BIPAP were intubated (*P* = 0.003). The average time to intubation was 124 (15–510) minutes in the intubated group. The mean time to intubation in neonates with HFNC was 159 ± 131 min and in neonates with NCPAP or BIPAP was 70 ± 78 min (*P* = 0.016).

### Model evaluation

**Figure 4:**
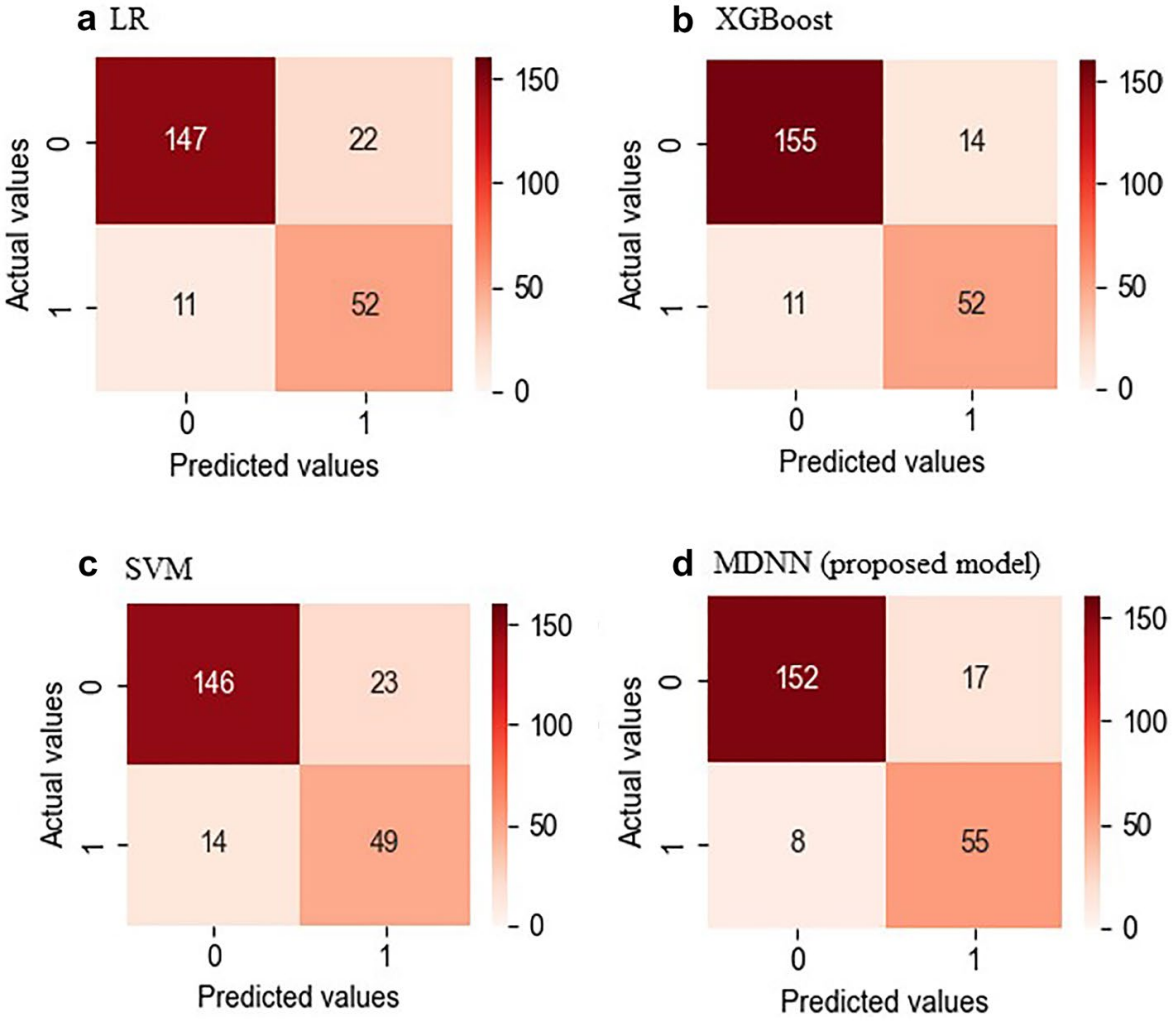
Confusion matrices for the entire dataset. (**a**) Linear regression (LR), (**b**) extreme gradient boosting decision tree regressor (XGBoost), (**c**) support vector machine (SVM), (**d**) multimodal deep neural network (MDNN) (0, non-intubation case; 1, intubation case).

**Figure 5:**
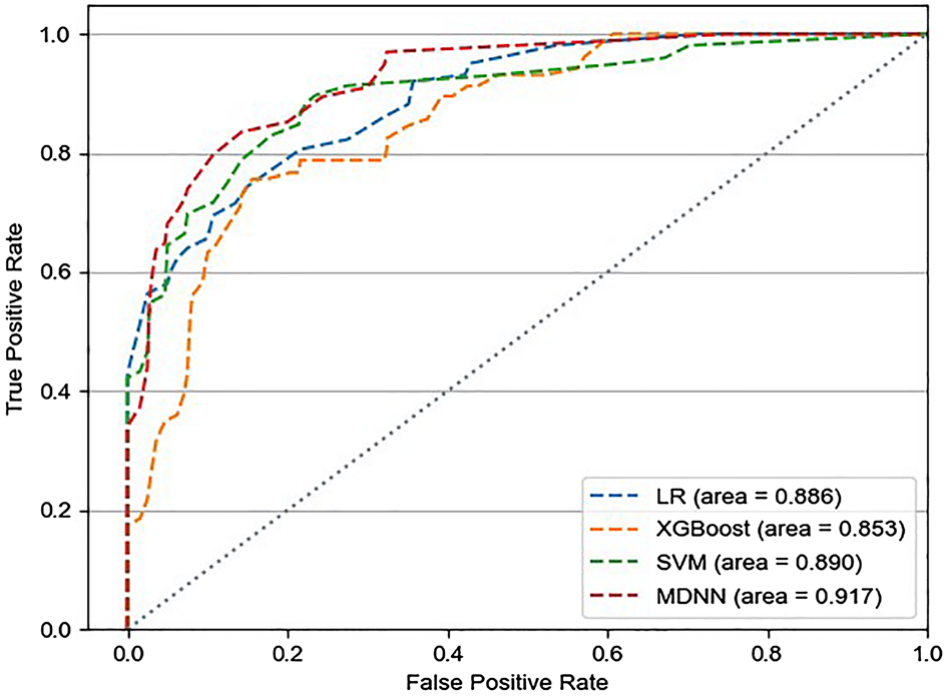
Area under the curve of receiver operating characteristics with fourfold cross validation. Linear regression (LR), extreme gradient boosting decision tree regressor (XGBoost), support vector machine (SVM), multimodal deep neural network (MDNN).

Figure 4 shows the confusion matrices of the entire dataset for each model. The MDNN and conventional ML (LR, SVM, and XGBoost) models were evaluated regarding the mean AUROCs and confusion matrices from fourfold validation (Fig. 5). The average AUROCs for these models were 0.917 for MDNN, 0.890 for SVM, 0.886 for LR, and 0.853 for XGBoost. In addition, the MDNN outperformed the ML model with respect to four metrics (F1-score, sensitivity, specificity, and accuracy). Specifically, the MDNN showed the best performance with F1-score of 0.884, sensitivity of 85.2%, specificity of 89.2%, and accuracy of 88.2%, followed by the SVM model with an F1-score of 0.882, sensitivity of 82.7%, specificity of 89.7%, and accuracy of 88.0% (Table 2).

**Table 2:** Comparison of model performances between the proposed model and the conventional machine learning models.

Model	Mean (SD)				
	AUROC	F1-score	Sensitivity, %	Specificity, %	Accuracy, %
LR	0.886 (0.081)	0.839 (0.093)	83.4 (6.1)	83.2 (13.3)	83.3 (9.8)
XGBoost	0.853 (0.069)	0.810 (0.094)	79.5 (7.7)	80.5 (11.8)	80.5 (9.9)
SVM	0.890 (0.052)	0.882 (0.052)	82.7 (6.0)	89.7 (7.4)	88.0 (5.4)
MDNN	0.917 (0.042)	0.884 (0.048)	85.2 (9.3)	89.2 (6.8)	88.2 (5.0)

*LR* linear regression, *XGBoost* extreme gradient boosting decision tree, *SVM* support vector machine, *MDNN* multimodal deep neural network, *AUROC* area under the curve of receiver operating characteristics.

### Model interpretation

To interpret the proposed model prediction, we used Shapley Additive Explanations (SHAP)^24^ and sensitivity analysis representing the contribution of each feature to the model outcome. A positive SHAP value indicates that the corresponding feature contributes to a higher probability of needing intubation, whereas a negative value suggests that the corresponding feature leads to a lower probability of requiring intubation. The magnitude of the SHAP value represents the contribution of a feature to prediction performance.

**Figure 6:**
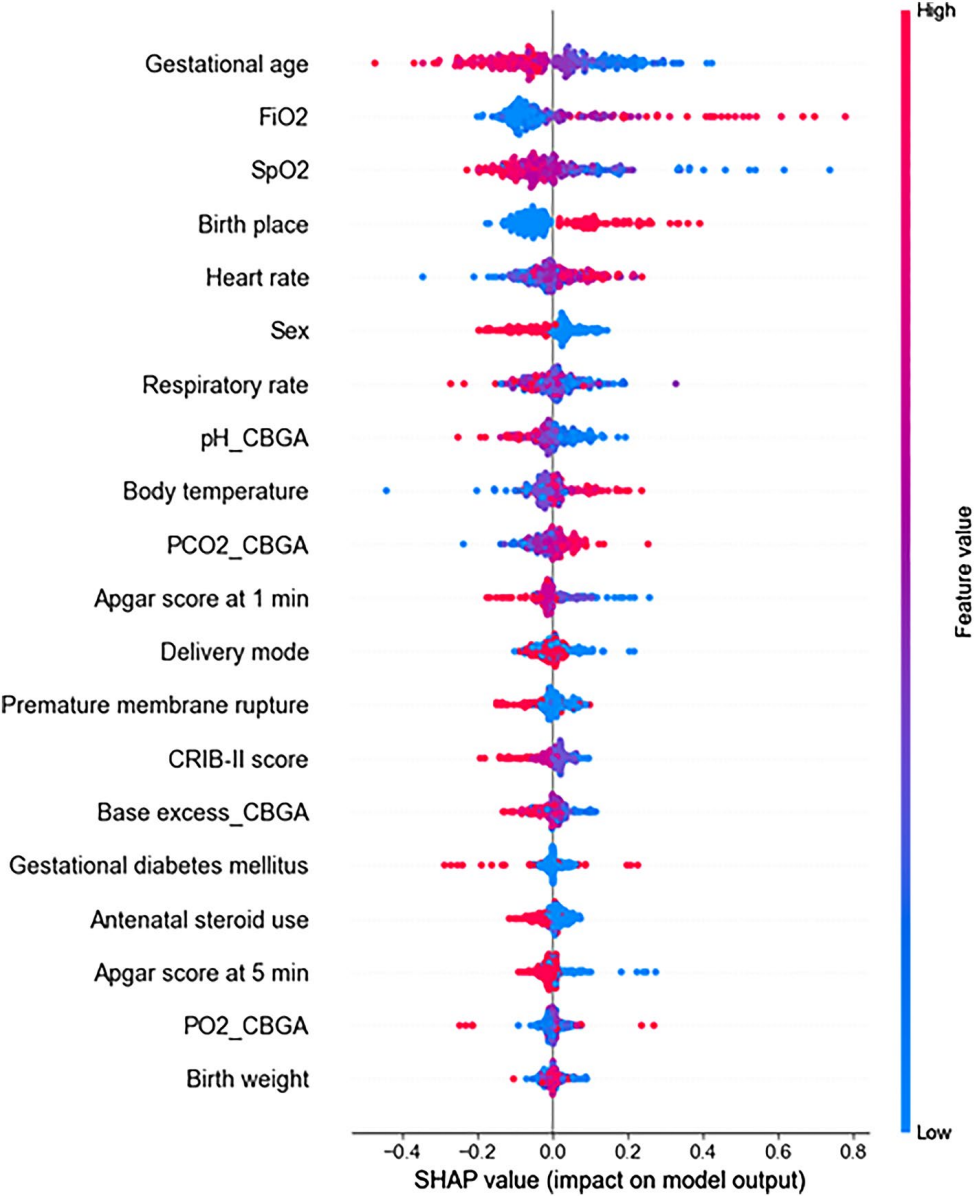
Feature importance assessment with Shapley additive explanations (SHAP) values. The most crucial features associated with the predictive power of the model were gestational age, fraction of inspired oxygen, pulse oximetry, birthplace, and heart rate.

Figure 6 shows a summary plot of the SHAP values used to visualize model interpretation. These results showed that GA, FiO_2_, SpO_2_, birth place, and HR were identified as the key features of the MDNN model. In addition, we performed additional SHAP analysis for the three machine learning models. In the LR model, the top five factors associated with intubation risk were GA, FiO_2_, birth place, BE in the initial blood gas analysis, and SpO_2_, and those of SVM were FiO_2_, SpO_2_, HR, and GA (Supplementary Fig. S1). The four important factors for these models were identical to those of the proposed model (GA, FiO_2_, SpO_2_, birth place, and HR). XGBoost showed that pH and BE in the initial blood gas analysis, birth place, SpO_2_, and GA were the important factors, which showed the greatest difference from the proposed model and the worst performance in AUROC and F1-score (Table 2).

**Figure 7:**
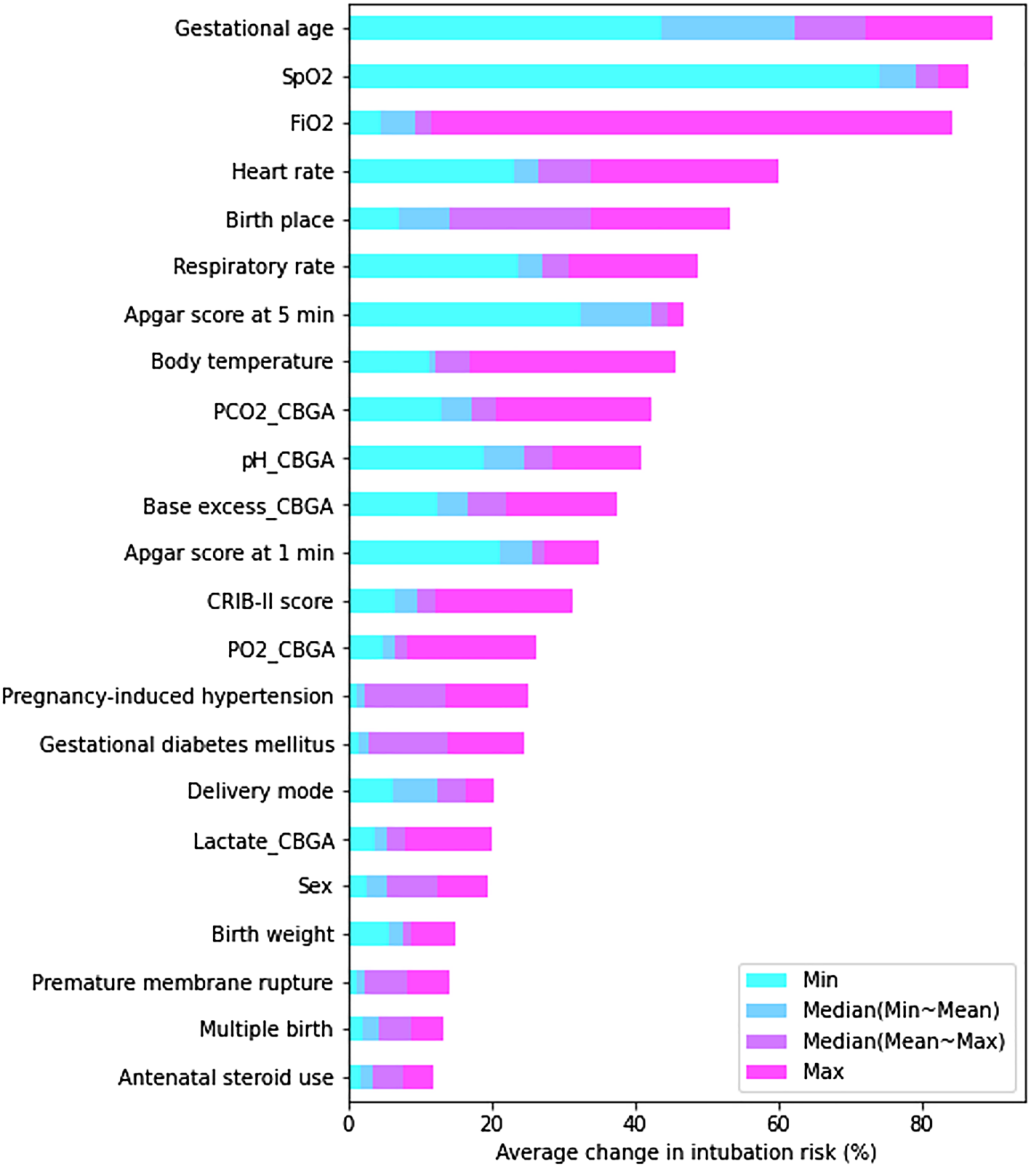
Sensitivity analysis for the intubation risk prediction of the multimodal deep neural network model. This chart showed the average changes in the intubation risk (%) due to varying each attribute individually while holding all others at their mean.

The sensitivity analysis was also performed as follows: We held all the attributes at their mean value while varying just one of the inputs to evaluate how input parameters affect the output variation derived by the proposed model. Five representative values (minimum, mean-to-minimum median, mean, mean-to-maximum median, and maximum) for each feature were used in this analysis. The baseline output was initially derived from the mean values of all features, and the changes in intubation risk (%) from baseline output were then calculated. The absolute values of the cumulative changes from the 23 features are plotted in Fig. 7. Figure 7 informed us that the GA causes the greatest change in the intubation risk (%), followed by SpO_2_, FiO_2_, HR, and birth place.

In both the sensitivity analysis and SHAP analysis, the top five factors were perfectly matched with slight differences in order. This result informed us that the GA causes the greatest change in the intubation risk (%), and the other key factors such as SpO_2_, FiO_2_, HR, and birth place also contributed significantly to the model prediction.

## Discussion

We collected datasets of 128 neonates with respiratory difficulties who underwent initial NIV therapy and developed an MDNN model to identify neonates requiring endotracheal intubation and mechanical ventilation within the following 3 h. The proposed model should provide useful information to alert medical staff of a need for intubation occurring within a short time (< 3 h) and reduce persistent monitoring efforts.

There were several studies on adult intubation^17,25,26^. Varzaneh et al.^25^ predicted the intubation risk of hospitalized coronavirus disease 2019 (COVID-19) patients using a decision tree-based model and showed a reasonable level of accuracy (93%). Siu et al.^17^ also predicted intubation in adults using a random forest model with an open dataset (medical information mart for intensive care, MIMIC) and achieved an AUROC of 0.87. As a study targeting NICU patients, Clark et al.’ study^27^ was conducted on very low birth weight infants (birth weight < 1500 g). Vital sign and electrocardiogram data collected at 2-s intervals were used to predict intubation after 24 h using a logistic model and had an AUROC of 0.84.

The MDNN, based on a multimodal approach, has the advantage of accessing multivariate information simultaneously, resulting in the highest predictive performance with an AUROC of 0.917, sensitivity of 85.2%, and specificity of 89.2%. The ablation study was performed to compare the non-time series model (tabular data model). We input only 19 tabular data to the deep neural network (DNN), and the structure of the DNN was constructed by removing the first MLP block that analyzes time-series data. The DNN achieved an average of AUROC 0.680, which decreased by 0.237 compared to the MDNN model. The other performance metrics of the DNN model were as follows: F1-score, 0.718; sensitivity, 69.5%; specificity, 70.5%; accuracy, 68.0%. These results showed that multimodal analysis was essential for improving performance.

In addition, we computed SHAP values to characterize the clinical factors potentially contributing to intubation in the MDNN model. SHAP values have been widely used to explain and clinically validate model outcomes^28–32^. The features with the highest SHAP values for the proposed model were GA, birth place, HR, FiO_2_, and SpO_2_. Lower GA and high fractional oxygen requirements have been considered clinically significant factors for RDS in previous studies^12,17,19,33–36^. For the machine learning models, the four key factors of SVM (GA, FiO_2_, SpO_2_, and birth place) and LR models (GA, FiO_2_, SpO_2_, and HR) were consistent with those of the proposed model. In addition, the XGBoost showed three important factors (GA, SpO_2_, and birth place), which showed the worst performance in AUROC and F1-score. From these results, it can be inferred that these five key factors (GA, FiO_2_, SpO_2_, birth place, and HR) from the MDNN model highly contribute to the model performance, and these factors were almost consistent across the study. The findings of this study suggest that by monitoring significant factors with the highest SHAP values, including time-series data like HR and SpO_2_, we would be able to predict a neonate who requires prompt endotracheal intubation. The findings of this study suggest that by monitoring significant factors with the highest SHAP values, including time-series data like HR and SpO_2_, we would be able to predict a neonate who requires prompt endotracheal intubation.

Our study was designed to predict intubation within 3 h using initial tabular data and time-series data collected over 1–3 time points recorded at 1-h intervals. Longer time-series data with dense intervals can help stabilize and improve model performance. However, among 36 neonates who underwent endotracheal intubation within 12 h, 29 neonates required endotracheal intubation within 3 h of admission. Therefore, prompt decisions must be made using short-term records. Furthermore, our dataset targeted neonates who initially received NIV supports, and NIV failure commonly occurred in the first-hour stabilization period; therefore, the short-term prediction (≤ 3 h) is the most practical for use in the NICU. In addition, the interval between recording time-series data varies from minutes to hours at each institute; therefore, this model can be practically applied to other situations. We used 23 clinical variables to predict the number of infants requiring endotracheal intubation and mechanical ventilation. Diverse data, such as radiologic images, could improve model performance. However, it is difficult to adjust radiologic image findings that require data labeling; therefore, we selected the minimum variables easily obtained at NICU admission as input variables. Furthermore, neonatal patients requiring intubation after 12 h were excluded. The tabular data included critical information such as body temperature and blood gas analysis results (pH, PCO_2_, PO_2_, BE, and lactate). Although these values could gradually recover or worsen over time, the initial data alone were collected, to alleviate the model’s complexity and improve its practical use. For long-term (> 12 h) predictions, we would try to input these data every hour.

RDS is the most common cause of respiratory distress in neonates who require endotracheal intubation within 48 h of birth^37–39^. Of the 36 neonates in the intubated group, 30 neonates with RDS and 2 with meconium aspiration syndrome received surfactant replacement therapy. In practice, the differential diagnosis of respiratory morbidities should be made using a combination of FiO_2_ to maintain normal SpO_2_, RR, degree of respiratory distress, and aeration of the lung on radiologic images^36,40^. Less severe respiratory diseases such as transient tachypnea of the newborn and mild RDS spontaneously resolve with NIV support within 48 h of life^37,38,41^. Delayed diagnosis of moderate to severe RDS leads to several complications including air leaks and intraventricular hemorrhage^9,42^. Timely diagnosis of RDS and earlier surfactant replacement therapy will improve neonatal outcomes. Several observational studies have attempted to predict RDS and NCPAP failure using perinatal variables such as prenatal ultrasound measures^43^, biomarkers in gastric aspirate^44,45^, and a combination of maternal and neonatal data at birth^46,47^. In this study, we developed a model to predict the need for endotracheal intubation within the next 3 h using 19 tabular data variables at birth and 4 time-series variables representing varying patient conditions before endotracheal intubation.

Since 2011, the insurance system in South Korea has covered early prophylactic surfactant administration in preterm neonates with a GA < 30 weeks or a birth weight < 1250 g within 2 h of birth^8^. In this study, 73 neonates who had already been intubated for prophylactic surfactant administration or for medical conditions requiring resuscitation within the delivery room were excluded. Therefore, most of the enrolled patients were late preterm or term neonates. During the study period, we implemented the conventional surfactant administration method (endotracheal intubation, bolus instillation with subsequent intermittent positive pressure ventilation for distribution, followed by mechanical ventilator support) instead of an intubation-surfactant-extubation (INSURE) strategy^48^ or a less invasive surfactant administration (LISA) technique^49^. Among the enrolled neonates, GA was still one of the significant factors predicting intubation within the following 3 h, although GA did not differ between the intubated and non-intubated groups. The intubated group showed significantly more severe respiratory acidosis than the non-intubated group. De Bernardo et al. have reported that low umbilical cord blood arterial pH (< 7.12) was correlated with RDS in full-term neonates^50^. Blood gas analysis is a useful tool for evaluating in patients with respiratory failure and severe respiratory acidosis is a definite indication for endotracheal intubation. Because endotracheal intubation is one of the most invasive procedures in the NICU, clinicians are reluctant to perform intubation in unclear cases. Moreover, pH and PCO_2_ were not considered among the top five factors in both SHAP and sensitivity analyses and were thus not included as key factors in our study. FiO_2_ and SpO_2_ were included in the five key factors and were already associated with RDS and CPAP failure according to the previous studies, even in the era of the LISA technique^51–54^. HR variability is known as a key factor for predicting mortality and sepsis in adults^55,56^ and neonatal medicine^14,57,58^.

The number of neonates assigned to NCPAP or BIPAP support was significantly higher in the intubated group than in the non-intubated group. The NIV failure rate in neonates receiving NCPAP or BIPAP was significantly higher than that in those receiving HFNC support. Additionally, the time to intubation was significantly shorter in the NCPAP and BIPAP groups than in the HFNC group. There could be a potential bias that physicians assigned neonates with more severe respiratory distress to NCPAP or BIPAP^3,59^. Artificial intelligence has recently expanded its clinical scope in modern medicine, especially for critically ill patients. With the aid of this artificial intelligence model, physicians’ performance can be more accurate and refined.

### Limitations

Despite the benefits of the artificial intelligence techniques applied here, our study had some limitations. First, the time-series variables were collected at 1-h intervals; therefore, data may be insufficient to capture all relevant clinical changes. Second, the model was evaluated using single-center data. Since we have no documented protocol defining when to intubate infants with respiratory difficulty, decision-making occurred on a case-by-case basis when NIV support was deemed insufficient. Third, there is limited open dataset for neonatal patients who experienced respiratory distress. The specific protocol for data collection makes it more difficult to find an equivalent dataset for comparison, and we could not perform model validation due to the insufficient number of positive cases (intubation) during training and testing. Therefore, our model requires external validation in other cohorts. Additionally, this study was designed to predict the need for short-term (≤ 12 h) endotracheal intubation in neonatal patients. For mid- to long-term predictions, we could technically improve the model architecture and reduce the time interval for clinical data after NICU admission. Furthermore, this model could be expanded and applied to the subacute and chronic hospitalization in the NICU. To our knowledge, our model is the first MDNN model to predict the need for intubation within the next 3 h in neonates with respiratory distress.

During the study period, all the world have experienced the pandemic era, fortunately, there were no COVID-19 related effects in this study. All pregnant women and newborns who were admitted to our institute underwent severe acute respiratory syndrome coronavirus 2 reverse transcription-polymerase chain reaction testing. None of the enrolled infants had COVID-19 infection.

## Conclusions

The integration of clinical data and time-series variables using MDNN predicted the need for intubation within the next 3 h in neonates with respiratory distress with 88.2% accuracy. The proposed model could help in the decision-making for neonates with respiratory distress who require endotracheal intubation. Further investigation is required to apply continuous time-series variables to the model and integrate the software into clinical practice.

## Supplementary Information

Supplementary Information.

## Data Availability

The data that support the findings of this study are available from the corresponding author (dalen@hanmail.net, dalen@chungbuk.ac.kr) upon reasonable request.
