# Supplementary material for: Predicting the need for intubation within 3 h in the neonatal intensive care unit using a multimodal deep neural network
Source: Sci Rep. 2023 Apr 17;13:6213. doi: 10.1038/s41598-023-33353-2 (PMC10106895; doi:10.1038/s41598-023-33353-2)
Supplement: Supplementary file 1 — Supplementary Information. [file 41598_2023_33353_MOESM1_ESM.pdf]

Predicting the Need for Intubation Within 3 Hours in the Neonatal Intensive Care Unit Using a Multimodal Deep Neural Network

Jueng-Eun Im, MS[1¶]; Seung Park, PhD[1¶]; Yoo-Jin Kim, MD[2]; Shin Ae Yoon, MD, PhD[2*]; Ji Hyuk Lee, MD, PhD[2]

[1]Biomedical Engineering, Chungbuk National University Hospital, Cheongju, Republic of Korea

[2]Department of Pediatrics, Chungbuk National University Hospital, Chungbuk National University College of Medicine, Cheongju, Korea

[¶]These authors contributed equally to this work.

Supplementary Data

Supplementary Table S1. A detailed description of each fold in our database

	Train		Test	
	**# Patients**	**# Sample cases**	**# Patients**	**# Sample cases**
**Fold 1**	97	167	31	49
**Fold 2**	96	154	32	41
**Fold 3**	96	139	32	87
**Fold 4**	95	155	33	55

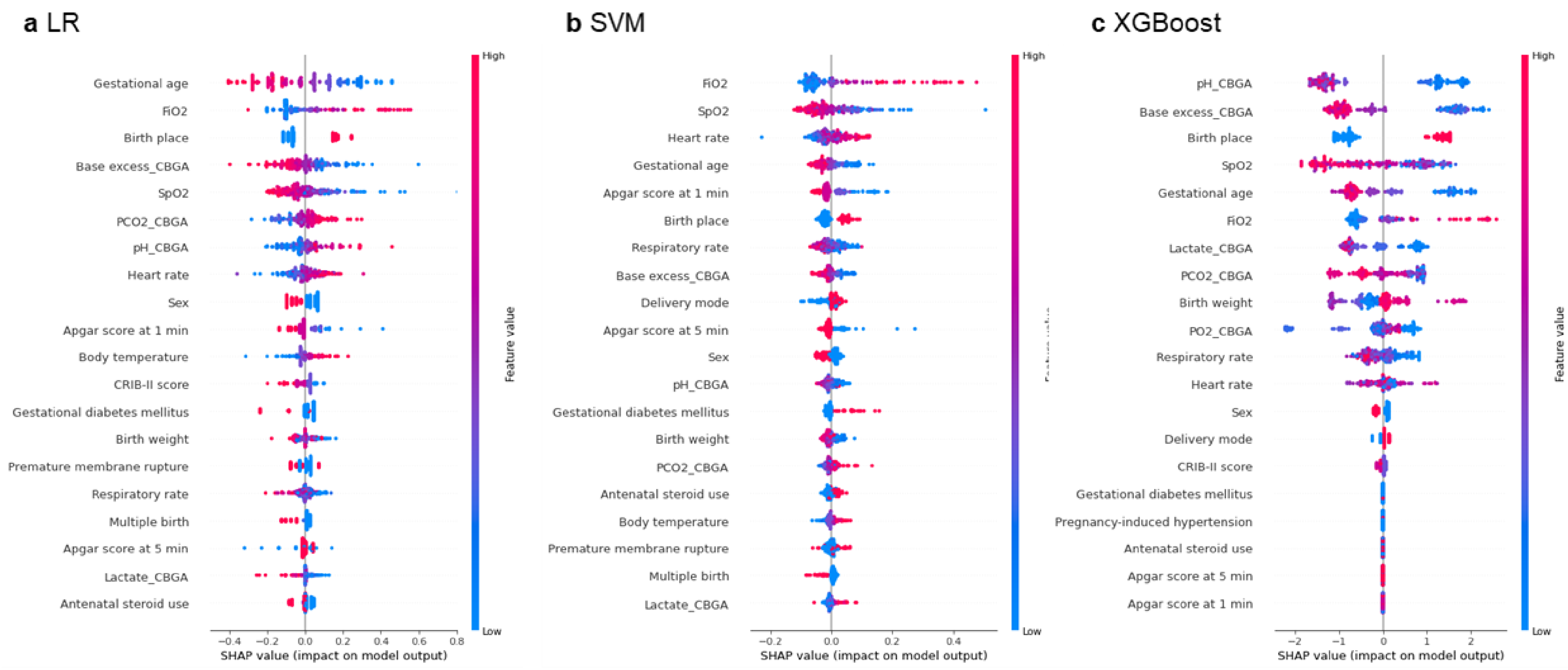

Supplementary Figure S1. Feature importance assessment with Shapley additive explanations values for the machine learning models.

a, Linear regression (LR); b, support vector machine (SVM); c, extreme gradient boosting decision tree regressor (XGBoost);
